# Development of a dynamic clinical assessment for finals

**DOI:** 10.1038/s41415-024-8028-x

**Published:** 2024-11-22

**Authors:** Francesca Mullan, Sarah Rolland, Hannah Desai, Simon James Stone, Heidi Louise Bateman

**Affiliations:** School of Dental Sciences, Faculty of Medical Sciences, Newcastle University, Newcastle upon Tyne, UK

## Abstract

Different dental schools assess ‘finals' in different ways. Assessment of applied clinical knowledge and skills are commonly based on either objective structured clinical examinations or multiple ‘long cases'. While iterative changes naturally occur throughout education and assessment, it is important to step back and question what skills should be assessed and either identify or develop new assessment methods. At the point of graduation, these should capture the professional attributes expected of a ‘qualified dentist'. New graduate attributes we consider valuable, which can often be challenging to assess summatively in a high-stakes examination, include effective communication skills in a variety of contexts, the ability to be reactive and responsive, and to manage case complexity appropriately for the graduate level.

Here, we report a dynamic assessment incorporating candidate interaction with a range of stakeholders. To reflect progression of changing clinical situations, we move away from solely traditional case-based discussions, which principally assess interaction with an examiner, and have introduced multiple scenarios with rapid situation changes, long-term follow-up, treatment complications and challenging communications. A cross-cutting assessment rubric was developed to assess candidates' information gathering/giving, communication, clinical and diagnostic reasoning, applied knowledge, management and professionalism.

## Key points

- Describes development and implementation of a dynamic multiple-scenario undergraduate assessment.
- Highlights challenges with different forms of clinical assessment.
- Considers quality assurance processes for assessments and the need to train those involved in delivery of a new assessment.

## Background

Final exit examinations in dentistry assess candidates' readiness for completion of degrees and entrance into largely independent clinical practice. These usually combine assessment of written knowledge and clinical skills components. At the point of graduation, assessment should capture the professional attributes the stakeholders expect of a newly qualified dentist, referred to in the UK as the ‘safe beginner'.^1^^,^^2^ However, following a consultation, the regulator of the profession, the General Dental Council (GDC), has recently replaced this with the concept of the ‘safe practitioner'.^3^

Undergraduate clinical examinations have focused mainly on the *viva voce* long case examination and objective structured clinical examination (OSCE) formats. While these are widely used and have an evidence base,^4^^,^^5^^,^^6^^,^^7^ they may not be the optimal assessments for students who are preparing to exit and face the complexities of independent clinical practice.^8^

### Long case

The ‘long case' originally used ‘real' patients, with candidates given a certain amount of time to take a history and carry out a clinical examination before an oral examination with examiner(s). Long cases are seen as having high validity as they can be designed such that they can examine more of a true-to-life scenario in greater depth. However, there can be variation in candidate experience, as not all candidates encounter the same patient. Even if the same patient was encountered, the experience could still vary, as the patient's responses could vary from candidate to candidate, or the clinical presentation could change throughout the session.^8^ A further concern and potential inconsistency of using ‘real' patients is the potential for inherent conscious and unconscious bias which may not have been fully mitigated against. There are challenges in the marking, as some aspects of a long case are often not fully observed (for example, taking a history). Observing this aspect of the examination would provide opportunities to observe candidates' behaviours and communication skills. Generally, the overall focus of the traditional long case examination is the interactions with examiners rather than interactions with patients.^8^^,^^6^^,^^9^ Evolutions in the long case format from ‘real' to ‘standardised' patients reduce assessment variability.^6^^,^^10^ However, ‘standardised' patients often result in either only patient records being presented, hence losing the element of patient interaction, or focused wholly on observing communication skills. Additionally, long cases may lack validity and reliability because the testing is generally of a very small sample of the overall curriculum, with often a small number of long cases and candidates only being assessed by a small number of examiners.

### Objective structured clinical examination

OSCEs have been extensively used in medicine, dentistry and wider healthcare professions since at least the 1970s.^4^^,^^7^ The OSCE has a number of short ‘stations', often between 5-10 minutes in duration, with the candidates rotating from one station to another. By the end of the examination, all candidates will have experienced all the stations. A typical examination will have around ten stations, which, unless carefully designed, may not be adequate to ensure reliability, with the total assessment time often being around one hour. Within each station, there is structured examiner and candidate information, with all candidates asked the same questions or instructed to perform the same procedure. Candidates are then often marked using a standardised checklist.

OSCEs have been praised for their reliability (repeatability), which can be increased with the inclusion of more stations and therefore overall testing time, but have been criticised for their inability to adequately assess communication skills or deeper level thinking.^10^^,^^11^^,^^12^ A further criticism of the OSCE's ability to adequately assess high-stakes examinations or complex clinical skills is the checklist used for grading. While checklists provide a standardised approach, the ability to pick up many marks yet miss something essential and still pass the station overall is a concern. Checklists may also encourage candidates to learn skills in stepwise fashion rather than holistically learning a skill.^13^ OSCEs have also been criticised for their relatively low validity due to the requirements of performing isolated tasks without necessarily integrating them into a clinical context; although, this can in part be offset by standard setting and incorporation of some actor-attributed marks.^14^ When checklists are compared with global scores in assessing complex skills, global scores are favourable, especially for seasoned examiners.^15^ However, rubrics are better for more complex scenarios assessed later in the course as, while they are standardised, their use of a scale allows them to better discriminate between weaker and poorer performances.^15^^,^^16^^,^^17^

Applied knowledge can be successfully assessed in both OSCEs and long cases and is essential in any clinical examination rubric. Clinical reasoning, however, is higher level thinking and not only encompasses applied knowledge, but combines the clinical skills of information gathering, communication, diagnostics and clinical judgement. This is a complex skill to assess and cannot adequately be assessed through a checklist. Holistic style matrices assess the candidate's overall performance in an encounter and are recommended for complex problem-solving types of assessments, such as clinical scenarios.^18^

OSCEs will continue to have a place in dental education and are a useful assessment tool for candidates in their middle years of training continuing their educational journey with further academic and clinical study, as the students will continue to develop their knowledge, skills and practical clinical experience. However, an OSCE may not adequately assess a candidate's ability to manage complex scenarios reflective of real-life encounters in clinical practice, which is required for final examinations.

### Longitudinal monitoring

It is important that appropriate patient safety thresholds are in place at the point of entry into supervised clinical practice and at the exit point towards independent practice. Longitudinal monitoring throughout a student's educative journey and development provides a portfolio of evidence that the student has met the required standard to progress onto the next stage of their career. Traditionally, monitoring was carried out with logbooks, which have evolved into comprehensive electronic portfolios containing detailed information about attendance and absence, procedures undertaken, grades obtained, in-course clinical assessments, case study portfolios, personal development plans and reflective diaries.^19^^,^^20^ A review of a student's longitudinal monitoring alongside reviewing evidence in relation to their professional behaviours can form the basis of a ‘sign-up' process, confirming readiness to enter the final examination.

An assessment should balance realism of clinical practice, and therefore validity, with repeatability, taking account of the reality that there are limits to the time and resources that are available to deliver assessment. Ideally, all parts should be observed and in a clinical assessment, which should incorporate any patient and non-patient based interactions, such as case-based discussions.^21^ However, it is not practical or possible for an examination to assess every possible interaction and situation a new dental graduate may face. The ‘finals' examination should be treated as an exit examination to ensure a candidate has demonstrated the transferable skills required to enter the dental workforce. The Newcastle School of Dental Sciences have a sign-up process to confirm candidates' readiness to sit the Stage 5 BDS (Bachelor of Dental Surgery) clinical finals exit examination. The sign-up is comprised of both in-course knowledge and clinical assessments. The knowledge components comprise a Dental Public Health report, professionalism essay and Ionising Radiation (Medical Exposure) Regulations multiple-choice paper. The clinical components comprise the completion of in-course clinical assessments of competence, satisfactory clinical experience, paediatric case presentation, a restorative case portfolio, management of medical emergencies, and no ongoing clinical/professionalism concerns identified through monitoring processes, including behaviour review and fitness to practise.

## Multiple Observed Standardised Long Examination Record

Following a review of current examination practice and the attributes and limitations identified above, we looked to introduce a new assessment. The following describes the Multiple Observed Standardised Long Examination Record (MOSLER) examination applied to dentistry. It will be described in detail to allow reproducibility. This examination was borne out of a similar examination style that has been applied to medicine (MBBS; Bachelor of Medicine and Bachelor of Surgery) examinations at Newcastle University^22^^,^^23^ and has been successfully delivered simultaneously across centres regionally and internationally.

The dental MOSLER has been developed to combine the attributes of the established examination styles capable of assessing the complex higher-level thinking (long case examination) with the reliability of an OSCE examination. There are similarities to the Objective Structured Long Examination Record (OSLER), which was originally proposed in medicine to help overcome shortcomings of long case examinations.^6^^,^^24^ The original OSLER consisted of ten items - four on history, three on physical examination, and the remaining three covering investigation, management and clinical acumen.^6^ The OSLER's effectiveness in assessing communication and complex skills has been recognised across different healthcare professions.^25^ However, questions have been raised about its practicality, as original recommendations suggested ten separate cases, requiring 20 examiners with a station time of 20-30 minutes.^6^ Other authors have evidenced that reducing the examination time is acceptable and that using two examiners offers little improvement to reliability.^21^ Therefore, our MOSLER builds on this, using eight stations with clearly defined objectives, a detailed global score domain-based marking scheme, and experienced examiners who have undergone station-specific training before each examination diet.

The primary task in developing new assessments is to identify what specifically is intended to be assessed - it should demonstrate relevance in terms of the skills being assessed and content validity to ensure it matches the appropriate learning outcomes.^26^ It is clearly not achievable for an assessment to test every clinical scenario a new graduate may encounter; however, complex scenarios are not only more realistic of clinical practice but can also cover broader aspects of a blueprint.^27^^,^^28^ When marking more involved clinical scenarios, holistic rubrics may be more appropriate than checklists, offering greater construct validity and internal consistency.^13^^,^^29^ However, an effective marking rubric requires adequate descriptors to assist the examiner to differentiate between what is expected of a newly qualified dentist, what fails to meet that standard and what is above that standard.^15^^,^^28^

In the context of a newly qualified dentist in the UK, many following graduation will enter a year of foundation or vocational training with a level of supervision.^1^ Therefore, in the development of the grading rubric, the standards set were based upon the level of supervision expected if the candidate were to encounter that scenario based upon their performance. In determining the criteria, four domains were identified, representing expectations of dentists by stakeholders^1^^,^^30^^,^^31^ (Table 1):

**Table 1:** MOSLER grading rubric

	Skills demonstrated AT or ABOVE the level of a safe beginner		Skills demonstrated that are NOT at the level of a safe beginner	
	Grade 4 Would require no supervision in encounter as a foundation dentist	Grade 3 Would require standard level of supervision in encounter as a foundation dentist	Grade 2 Would require more supervision than usual in encounter as a foundation dentist	Grade 1 Would require direct 1:1 supervision in encounter as a foundation dentist
Communication	Communicates effectively, sensitively, uses appropriate language and terminology Actively listens and responds, sharing ideas concerns and expectations Non-verbal communication appropriate eg positioning/body language	Competent communicator: at the lower end, there may be some hesitancy, occasional lapses in fluency and/or clarity Overall, terminology used does not impede understanding Respects ideas, concerns, and expectations Non-verbal communication does not detract from encounter eg position/body language	Significant lapses of fluency and/or clarity Frequent use of inappropriate/incorrect terminology used that risks or impedes understanding Acknowledges patients' ideas, concerns and expectations but may overly impose own ideas or views Little consideration of non-verbal communication eg position/body language	Little or no competence in use of communication skills, wholly or largely unclear Predominant use of inappropriate/incorrect terminology throughout or language eg swearing Impedes understanding Does not acknowledge or respect patients' ideas, concerns and expectations No consideration of non-verbal communication, distracting, actively detracts from encounter
Information gathering/giving	Engages in shared decision-making, gathers/provides the appropriate information to manage the encounter Succinct and well-structured history/interaction Significant facts elicited with minimal irrelevant detail	Gathers/provides sufficient information to manage the encounter, there may be some omissions Structured history/interaction with minor lapses of organisation Some deficiencies/repetitions/irrelevant details acceptable	Gathers/provides partial information but with significant omissions, understanding may be impeded Structure of history/interaction lacks Numerous repetitions or irrelevancies	Gathers/provides insufficient information to manage the encounter Most significant facts missed, major deviations from line of enquiry Unstructured/disorganised history/interaction
Clinical and diagnostic reasoning, applied knowledge, management	Important and relevant conditions in differential diagnosis supported by comprehensive and rational explanation Justified investigative strategy, does not request irrelevant tests Clear and logical planning supported by sound knowledge and clinical judgement Understands the implications of medical and social history Manages/recommends clinical procedures confidently, safely and efficiently, offers excellent standard of care	Important and relevant conditions in differential diagnosis Appropriate investigative strategy, although this may not be optimised (eg an irrelevant test or some omissions) Appropriate planning supported by knowledge and clinical judgement, some guidance and prompting may be required at the lower end Aware of the implications of medical and social history Manages/recommends appropriate and safe clinical procedures	Some omissions from the differential diagnosis Deficiencies in investigative strategy (eg multiple irrelevant tests/several important omissions) Deficiencies in establishing an appropriate plan, lacks clinical reasoning or justification Medical/social history noted but deficiencies in implications Manages/recommends clinical procedures that are not always appropriate eg under or overtreatment	Omits likely or ‘must not miss' diagnoses Major errors in investigative strategy that would make correct diagnosis unlikely and/or may result in potential harm to the patient Unable to treatment plan. No justification or clinical reasoning. Lacks knowledge and clinical judgement No consideration of medical and social history or its implications Manages/recommends clinical procedures that are inappropriate or unsafe - likely to result in patient harm
Professionalism	Professional behaviour exemplary, decisive, confident, encourages patient/staff confidence and safety Consistently recognises and acts within professional standards, relevant laws, and guidance Respects confidentiality, aware of need for valid consent and their impact on patient management Demonstrates effective team working and insight into multidisciplinary team Actively reflective of own limitations, able to identify and action appropriate onward referral in patient's best interests	Professional behaviour that maintains patient/staff confidence and safety Recognises and generally acts within professional standards, relevant laws, and guidance Respects patient confidentiality, aware of consent issues and that these may impact upon patient management Awareness of their role in multidisciplinary team, their own limitations, able to action an appropriate onward referral	Inconsistent professional behaviour, may impact upon patient/staff confidence Breaches professional standards, laws, or guidance Aware of need for consent but unsure as to necessary level or the impact on patient management Limited or inconsistent approach to working with a multidisciplinary team, lacks awareness of own limitations and need for appropriate onward referral	Inappropriate/unprofessional behaviour, impacts up on patient/staff confidence and/or safety Does not act at all within professional standards, laws, or guidance Acts without consideration, lacks insight into own behaviour Unaware of consent issues or impact on patient management Unaware of role in multidisciplinary team Unaware of own limitations and need for appropriate onward referral

- Communication
- Information gathering/giving
- Clinical and diagnostic reasoning, applied knowledge, management
- Professionalism.

Four grades are applied within each domain (1-4):

- Grade 4 - would require no supervision in the encounter as a foundation dentist
- Grade 3 - would require standard level of supervision in the encounter as a foundation dentist
- Grade 2 - would require more supervision than usual in the encounter as a foundation dentist
- Grade 1 - would require direct 1:1 supervision in the encounter as a foundation dentist.

The rubric is colour-coded to act as a visual aide for the examiners.

The dental MOSLER comprises eight ‘clinical encounters' which take place over a two-day period. The combined results determine a candidate's overall clinical examination outcome for finals. Each clinical encounter is 20 minutes long, including five minutes of reading. The encounters are comprised of:

- Three discipline-specific clinical scenarios (restorative dentistry [RD], paediatric dentistry [PD], oral and maxillofacial sciences [OMFSc]) using simulated patients (actors). Examiners observe the clinical encounter and may also have an active role, directly questioning the candidate at the end of the encounter
- Three discipline-specific (RD, PD, OMFSc) case-based discussions between the candidate and examiner. Clinical information may be provided sequentially to mimic a clinical encounter
- One interdisciplinary communication/professionalism scenario
- One interdisciplinary management of acute care (medical emergency scenario).

To ensure inclusivity and to minimise bias, all examiners and actors undertake conscious inclusion and equality and diversity training. Comprehensive information is given to the actors and examiners, including the standardised examiner questions for repeatability. Each student experiences the same scenarios; to achieve this, we have multiple tracks and concurrent clinical encounters running. A quarantine system is in place so candidates cannot interact with someone who has already completed a scenario. By the end of the MOSLER, each candidate is assessed by eight different examiners for a total of 160 minutes.

The grades are totalled for each domain, giving a maximum possible score of 32 (eight encounters x maximum domain grade of 4) for each domain. Candidates who are awarded grades below the ‘safe beginner' threshold (below Grade 3) may compensate for a low grade in a domain with a higher grade in the same domain in another clinical encounter elsewhere in the MOSLER.

The pass mark for each domain is 21 (minimum pass grades [Grade 3] achieved in five out of the eight stations and potential for three stations at non-passing grades [Grade 2]; 15 + 6 = 21). The merit mark is 29 (minimum pass grades [Grade 3] achieved in three out of the eight stations, and five higher passing grades [Grade 4] in the remaining five stations; 9 + 20 = 29). To achieve an overall ‘merit in clinical skills' award, a merit must be achieved in three out of the four domains, and a distinction award requires a merit in all four domains. Examples of possible passing and outcomes from the grade boundaries are shown in Table 2 and Table 3.

**Table 2:** Example of an illustrative passing outcome from MOSLER grade boundaries. The ‘pass mark' for each domain is 21 (minimum pass grades [Grade 3] achieved in five out of the eight stations and potential for three stations at non passing grades [Grade 2]; 15 + 6 = 21). The ‘merit pass' is 29 (minimum pass grades [Grade 3) achieved in three out of the eight stations, and five higher passing grades [Grade 4] in remaining five stations; 9 + 20 = 29). To achieve an overall merit in clinical skills award, a ‘merit pass' must be achieved in three out of the four domains and a distinction award requires a ‘merit pass' in all four domains. This illustrative passing candidate has been able to compensate for a single Grade 2 in information gathering achieved in their OMFSc case and a single Grade 2 in clinical and diagnostic reasoning achieved in their restorative case, with higher grades in other clinical encounters. While they achieved a single merit in the communication domain with a score of 30, the overall grade was a standard pass

Candidate A	Communication	Information gathering/giving	Clinical and diagnostic reasoning, applied knowledge, management	Professionalism
Paediatric actor	4	3	3	4
Paediatric case	4	3	3	3
Restorative actor	4	3	3	3
Restorative case	3	4	2	3
OMFSc actor	4	3	4	3
OMFSc case	4	2	3	4
Acute care	3	3	3	4
Communication	4	4	4	4
Total	30	25	25	28
Domain outcome	Merit	Pass	Pass	Pass
Overall outcome	Pass

**Table 3:** Example of an illustrative failing outcome from MOSLER grade boundaries. The ‘pass mark' for each domain is 21 (minimum pass grades [Grade 3] achieved in five out of the eight stations and potential for three stations at non passing grades [Grade 2]; 15 + 6 = 21).While this illustrative failing candidate was able to compensate for non-passing grades in the domains of clinical and diagnostic reasoning and professionalism by achieving higher grades in other clinical encounters, they were unable to achieve compensation in information gathering and giving. The pattern of failing grades in this domain would identify that the level of supervision was repeatedly below what would be expected and the illustrative candidate was not ready to pass finals

Candidate B	Communication	Information gathering/giving	Clinical and diagnostic reasoning, applied knowledge, management	Professionalism
Paediatric actor	3	2	3	2
Paediatric case	3	2	2	3
Restorative actor	3	4	4	3
Restorative case	3	1	2	2
OMFSc actor	3	2	3	3
OMFSc case	4	3	4	4
Acute care	4	4	3	4
Communication	3	2	3	2
Total	26	20	24	23
Domain outcome	Pass	Fail	Pass	Pass
Overall outcome	Fail			

In preparation for the introduction of the examination, a three-stage process of training was implemented. The first stage introduced the new format to examiners by providing mock-up video scenarios to illustrate the type of interactions the new examination included and promote familiarity with the new grading rubric. The second stage was a mock examination to familiarise examiners and students with the examination process, including examiner briefings, the types of clinical encounters, timings, quarantine arrangements and the grading rubric. The third stage was individual, clinical encounter-specific training, using videos of the scenarios which would be used for the examination.

The videos not only helped with examiner and actor alignment^32^^,^^33^ but they also offered insight into how scenarios may perform and allowed modifications to improve interaction timing or flow. We continue to undertake extensive station-level examiner and roleplayer training before each diet of the examination to maximise reproducibility across multiple examination tracks. The external examiners' reports from those who had experience of the previous established examination format and the new MOSLER were extremely positive. They provided a level of quality assurance that the new format MOSLER delivered what was intended and that implementation addressed concerns surrounding long-case and OSCE examinations.

The GDC's recent transition from ‘safe beginner' to ‘safe practitioner' will require us, in future, to further develop the MOSLER examination.

## Conclusion

The dental MOSLER has been undertaken successfully since 2019 and has been well-received from external examiners since its introduction. While all examinations have attributes and limitations, we would propose that the dental MOSLER is a useful examination for assessing the complexities of a clinical finals examination.

## Data Availability

Data sharing not applicable to this article as no data sets were generated or analysed.
